# Experimental Study of High-Pressure Oxy-Fired Direct Contact Steam Generation (HiPrOx-DCSG) with Steam-Assisted Gravity Drainage (SAGD) Produced Water

**DOI:** 10.1021/acsomega.5c09345

**Published:** 2025-11-20

**Authors:** Lijun Wu, Ted Herage, Mohammad Asiri, Bruce Clements

**Affiliations:** Natural Resources Canada, 353130 CanmetENERGY in Ottawa, 1 Haanel Drive, Ottawa K1A 1M1, Canada

## Abstract

Direct contact steam generation (DCSG) produces steam-rich gas by directly contacting combustion gases with sprayed water. This water is typically produced water (i.e., recovered condensate) from a process that has already extracted heat from the steam-rich gas. DCSG enables the reuse of produced water for steam generation without the extensive treatment required by conventional boiler systems, making it suitable for applications where a high steam purity is not essential. Key challenges in DCSG include managing impurities during combustion and matching the flue gas pressure with the elevated downstream process pressure, often necessitating pressurized combustion. This requirement becomes advantageous when integrated with carbon capture and storage (CCS), especially with oxy-fired combustion where flue gas, following H_2_O condensation, yields a pressurized CO_2_-rich stream requiring less energy for downstream capture and compression. This study tested a pilot-scale high-pressure oxy-fired (HiPrOx) DCSG system operating at pressures of 80, 55, and 30 barg and temperatures between 1000 and 1250 °C, using steam-assisted gravity drainage (SAGD) produced water. The system successfully generated a pressurized steam-rich gas containing approximately 90% steam, balanced by CO_2_ and trace gases, suitable for CO_2_ coinjection into SAGD wells. Oxy-fired combustion effectively removed solids and impurities from the SAGD water; however, burner surface deposition was severe at 80 barg. Deposition was significantly reduced and acceptable at 55 barg and minimal at 30 barg. The results demonstrate the feasibility of HiPrOx-DCSG for utilizing highly contaminated water for steam generation.

## Graphical abstract

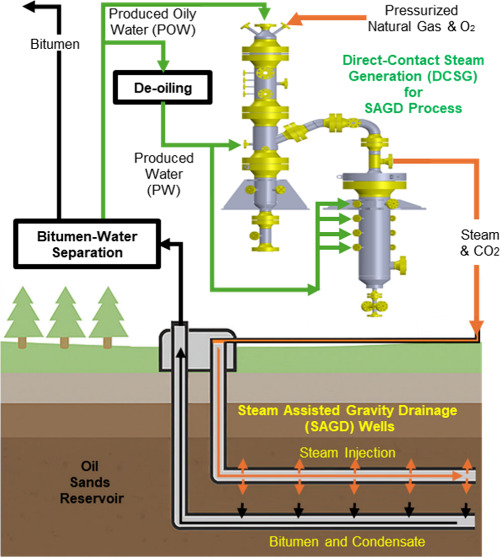

## 1 Introduction

Global CO_2_ emissions must reach net zero around 2050, and unabated fossil fuel use must be nearly eliminated by that time.^1^ In other words, fossil fuel use must decline sharply after 2025 and be largely phased out by midcentury, unless paired with carbon capture and storage (CCS).

Many industrial processes rely heavily on steam as a source of thermal energy for heating. The conventional method of steam generation uses a boiler system, where fossil fuel is combusted to indirectly convert water into steam through heat exchangers. This steam is then distributed through piping networks to deliver heat to target process streams, either via heat exchangers or through direct contact. Industries that commonly use steam in this way include power generation, oil and gas production, chemical and petrochemical manufacturing, pulp and paper, food and beverage processing, textiles, pharmaceuticals, mining, and district heating systems.

Ongoing efforts and technological advancements have been aimed at producing steam with less fossil fuel consumption, facilitating the transition to a low-carbon future in power and industrial sectors. Integration of renewable energy sources into steam production processes has gained increasing attention. Concentrating solar-thermal technologies, coupled with thermal energy storage technologies, will help reduce dependency on fossil fuel.^2^ Geothermal energy presents a promising approach to decarbonizing industrial steam production, especially advancements in deep geothermal technologies, such as enhanced geothermal systems, tapping into higher temperatures.^3–5^ Biomass enables steam generation without direct combustion of fossil fuels.^6^ In addition, electrifying the heat supply for industrial processes, using electricity as the major energy source rather than fossil fuels, offers an alternative pathway to reducing greenhouse gas emissions. Among the emerging electrifying technologies, high-temperature industrial heat pumps and mechanical vapor recompression are found to be the most dependable and high-performing technologies in a wide range of applications, compared to plasma technology and microwave and radio frequency heaters.^7,8^

Using less fossil fuel to reduce GHG emissions from industrial processes tackles only one of the issues associated with steam production. Another issue is the water used for steam generation. To minimize freshwater usage, condensates from industrial steam-heating processes are typically recycled and reused. In direct-contact heating with process fluids, the condensate becomes contaminated by process fluids and requires intensive treatment before it can be fed back into industrial boilers or renewable heating systems. Inappropriate feedwater treatment results in significant operating failures, especially for industrial boilers. The water treatment process in this context plays a decisive role in industrial processes.^9^ The water treatment process often involves multiple stages, such as filtration,^10,11^ chemical dosing, softening, reverse osmosis,^12^ and, in some cases, thermal stripping, to meet feedwater quality standards. These processes can be energy-intensive and costly and generate secondary waste streams, which must also be managed. For instance, in the oil sands steam-assisted gravity drainage (SAGD) extraction process, SAGD produced water is a complex mixture and emulsion of oil and water with a high concentration of dissolved organic matter.^13^ The once-through steam generators (OTSGs) used in existing SAGD extraction processes typically achieve steam quality of 80%, with 20% blowdown.^14^ For such processes, the production of the necessary quantities of steam may result in the depletion or serious reduction of locally available freshwater supplies, such as rivers or lakes, while secondary contaminated water sources are created by wastewater treatment. Consequently, sustainable steam generation must consider both energy and water footprints to effectively reduce the environmental impacts and operational costs.

In contrast to conventional boiler systems, direct contact steam generation (DCSG) produces a steam-rich flue gas through the direct contact of combustion gases with sprayed water. The sprayed water can be industrially produced water with minimal treatment or without treatment.

DCSG combustion can occur either in an air-fired environment^15^ or an oxy-fired environment.^16^ Known methods of DCSG using air-firing, when compared with conventional boiler steam generation, have the disadvantages of producing only a low concentration of steam due to dilution by the presence of a large quantity of nitrogen. Instead, the oxy-fired approach can provide a high concentration of steam balanced mainly with gaseous CO_2_. This is advantageous in the context of carbon capture and storage (CCS) because after steam has been condensed a highly concentrated CO_2_ stream can be produced, thereby simplifying downstream CO_2_ processing. Additionally, when an industrial process is at an elevated pressure, pressurized oxy-fired combustion is required, resulting in a highly concentrated CO_2_ stream at elevated pressure that can further reduce the energy requirements for downstream CO_2_ compression. Advancements in oxy-fired combustion technology have been mainly investigated for power generation with CCS.^17^ A high pressure oxy-fuel (HiPrOx) combustion process for power generation has been purposely designed at 80 barg in order to produce a supercritical CO_2_ stream that can be liquefied by room temperature cooling. A pump can then be used to increase the pressure of the liquid CO_2_ to meet pipeline requirements.^18,19^ The oxy-fuel combustion-based CCS configurations are relatively cost-effective, especially at high CO_2_ removal rates exceeding 90%, compared to traditional air combustion methods.^20^ Other industrial sectors with a strong potential to implement oxy-fuel combustion for CO_2_ capture include the lime and cement industry^21^ and the aluminum melting sector.^22^

From the water perspective, HiPrOx-DCSG found its application in the SAGD process.^23^ Figure 1 shows an existing SAGD process.^24^ Steam generated from a once-through steam generator (OTSG) is injected into a deep underground bitumen reservoir to heat the bitumen, reduce its viscosity, and enable the resulting mixture of bitumen and condensate to be pumped to the surface. After separation from the bitumen, the condensate – referred to as produced oily water (POW) – is deoiled to yield produced water (PW). PW typically contains high levels of silica (150–400 mg/L as SiO_2_), calcium hardness (5–150 mg/L as CaCO_3_), magnesium hardness (5–75 mg/L as CaCO_3_), total dissolved solids (TDS, 1000–2500 mg/L), and total organic carbon (TOC, 150–800 mg/L as C).^25–27^ To meet OTSG boiler feedwater (BFW) requirements (SiO_2_ < 50 mg/L, total hardness <0.5 mg/L as CaCO_3_, and oil and grease <0.5–1 mg/L), PW is first blended with fresh makeup water and recycled boiler blowdown (BBD) and then subjected to a water treatment process. Conventional processes use warm or hot lime softening (WLS/HLS) with chemical additions such as lime, soda ash, magnesium oxide, coagulants, and flocculants to reduce turbidity, silica, and hardness. Subsequent filtration and weak-acid cation exchange remove suspended solids and residual divalent ions, producing BFW suitable for OTSG operation.^25–27^ Although OTSGs can tolerate relatively high TDS (8000–12,000 mg/L) and TOC (300–1000 mg/L), they produce steam of relatively low quality, leading to boiler blowdown representing about 20% of the BFW.^28^ A portion of this blowdown is recycled to the softening step, while the remainder requires disposal. As a result, considerable expense is involved in treating the produced water that is extracted along with the bitumen to produce a sufficiently clean feedwater for the steam generator. SAGD plants can be considered to be water treatment plants with bitumen as a byproduct,^29^ making water treatment a major economic driver in SAGD operations.

**Figure 1:**
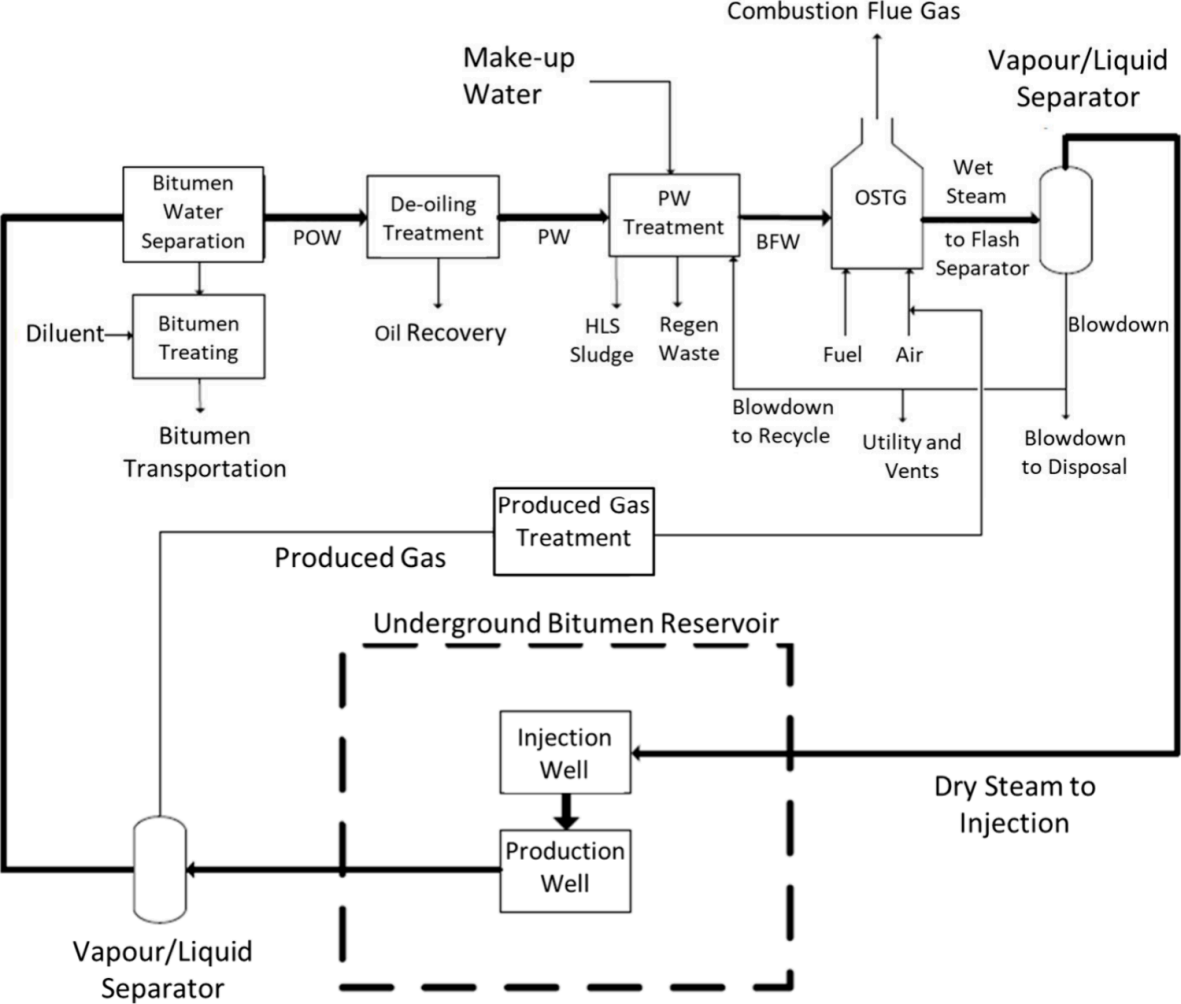
Existing SAGD process diagram, reprinted with permission from ref 24.

In an oxy-fired DCSG-SAGD process^24^ as shown in Figure 2, the SAGD POW and PW are directly sprayed into the DCSG for steam generation. The produced steam-rich CO_2_ mixture is then injected directly into the SAGD reservoir. This allows the reservoir to potentially retain CO_2_ for the sequestration of CO_2_ after steam condensing. Adding noncondensable gas (NCG) to the steam injection into the SAGD well was found to be beneficial by improving the thermal efficiency of the SAGD process.^30^ Common NCG candidates are methane, nitrogen, hydrogen, oxygen, carbon monoxide, carbon dioxide, as well as mixtures of these components; in field applications methane coinjection has been practiced since 2013.^31,32^ CO_2_ coinjection with steam has been investigated and found to have the potential to not only improve SAGD efficiency but also reduce greenhouse gas (GHG) emissions by CO_2_ storage.^33,34^

**Figure 2:**
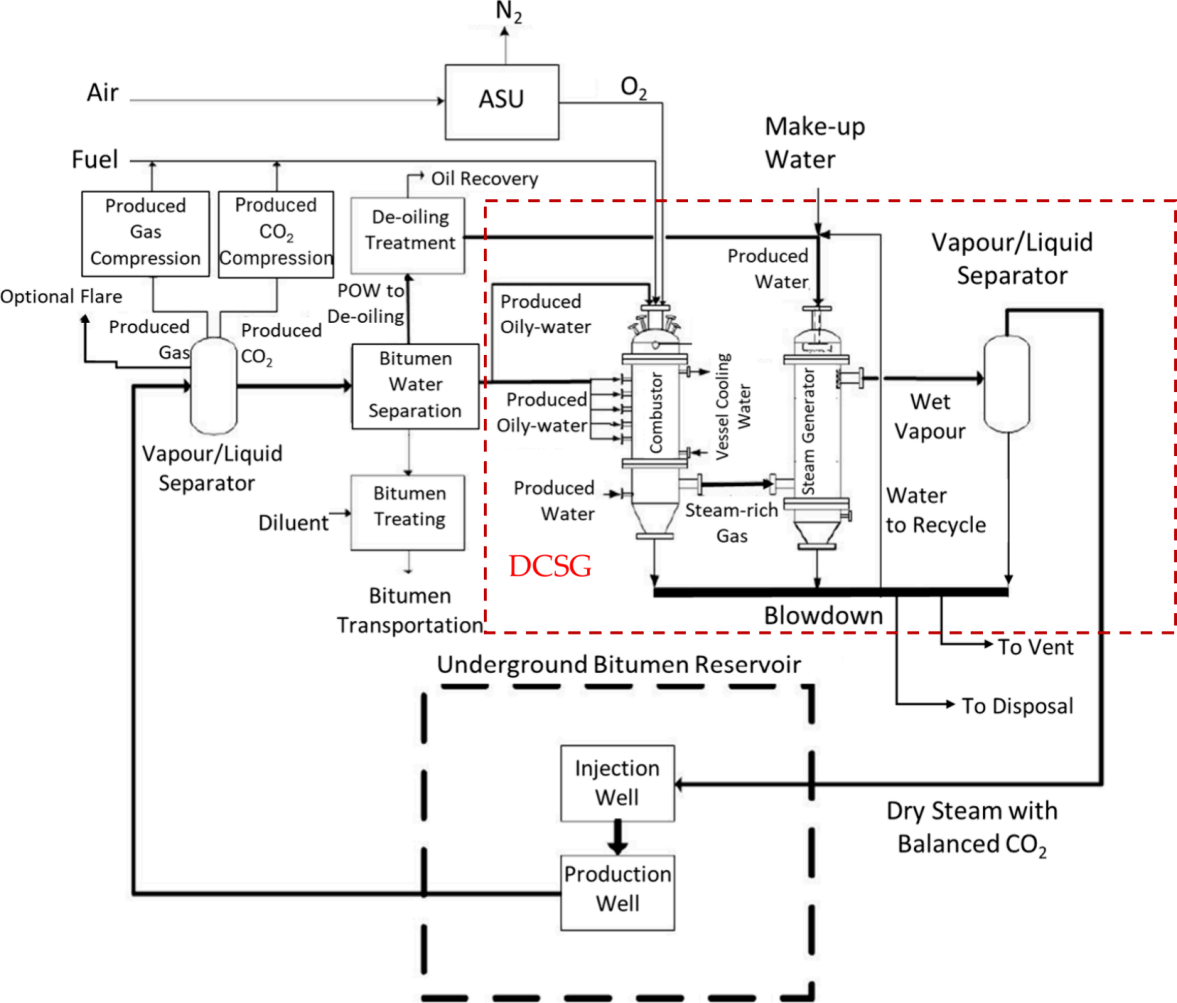
DCSG-SAGD process, reprinted with permission from ref 24.

The concept of HiPrOx-DCSG was first explored at CanmetENERGY in Ottawa through thermogravimetric analysis experiments at atmospheric pressure to study the effects of steam on the combustion of lignite coal char under oxy-fired conditions (O_2_/CO_2_/H_2_O). The results showed that increasing the steam content led to a reduction in both the burning time and combustion temperature.^24^ Subsequently, the DCSG concept was demonstrated using a pressurized combustion facility operating at 15 barg with city water (CW). The test fuels included *n*-butanol, graphite–water slurry, and their mixtures, to evaluate the applicability of the DCSG process to fuels with varying contents of volatile matter. The 15-barg DCSG experiments and results confirmed that a steam content of approximately 90 mol % (at saturation) in the combustion gas could be achieved, while maintaining ignition and stable combustion even in a high-moisture environment.^35^

The study in this paper investigated a pilot-scale HiPrOx-DCSG facility with SAGD water to evaluate the applicability of DCSG for SAGD processes. The SAGD produced water included the free water from the knockout tank outlet, which is hereby termed produced oily water (POW), and the induced static flotation tank outlet water, hereby termed produced water (PW), both from a SAGD facility in the Athabasca region of Alberta, Canada.

## 2 Pilot-Scale HiPrOx-DCSG Plant Overview and Methods

The pilot-scale HiPrOx-DCSG system shown in Figure 3 consists of three sections housed in two vessels. The first section is a reactor where oxygen, natural gas, and POW are simultaneously introduced through a burner, producing an effluent gas stream at temperatures exceeding 1000 °C. These elevated temperatures enable the dissolved and suspended solids in the POW to melt and flow down the reactor’s inner wall, while any residual hydrocarbons in the POW are fully combusted. The gas exiting the reactor contains approximately 90 vol % H_2_O.

**Figure 3:**
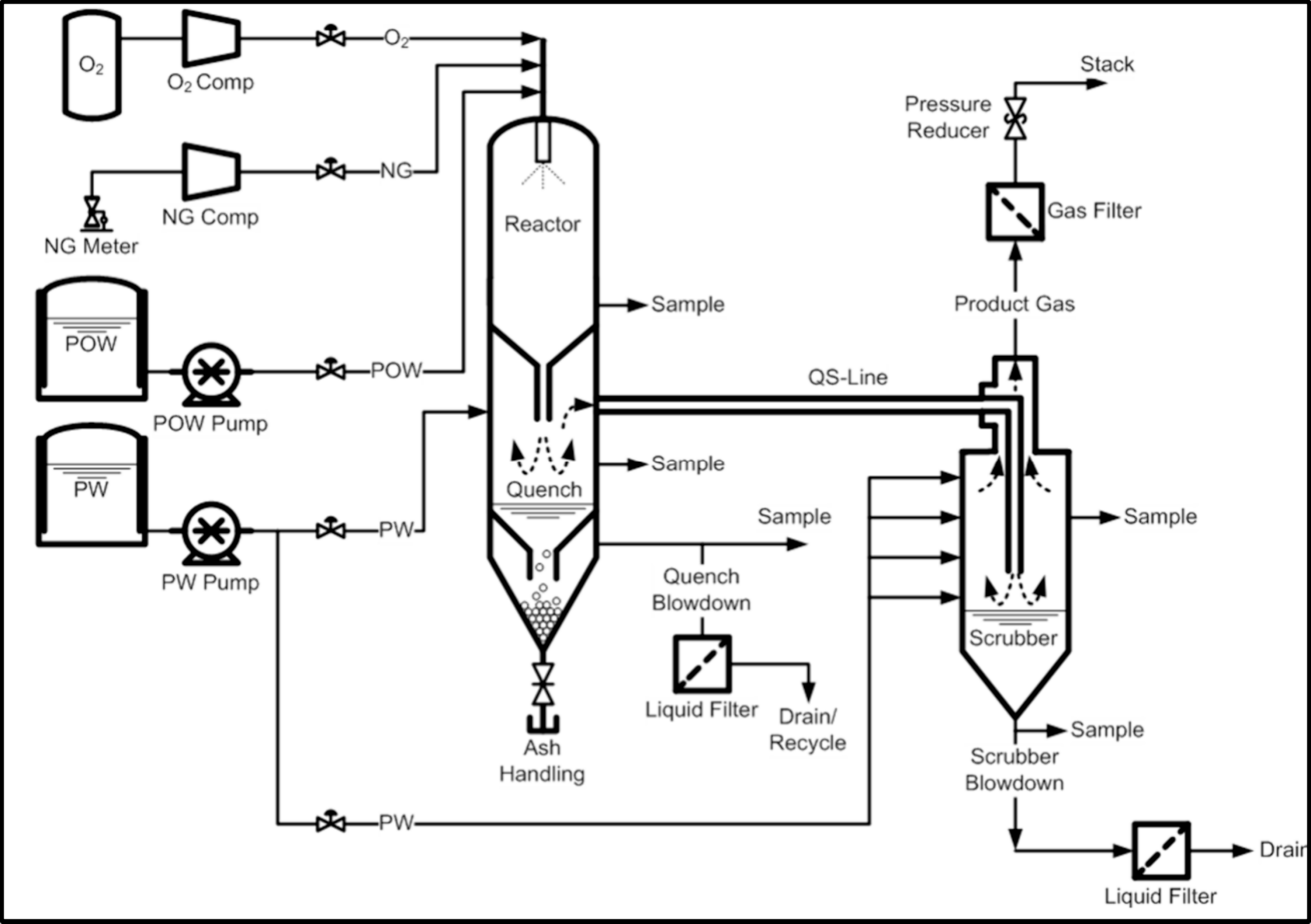
Simplified process flow diagram of the DCSG pilot plant.

Following the reactor, the gas enters a quenching section, where its steam content is further increased. This is achieved by passing the hot gas through an atomized spray of PW, reducing the temperature to 600–800 °C and increasing the steam content from 90 to 92 vol %. The quench also solidifies the mineral melt from the reactor walls, allowing the resulting solids to be removed via a blowdown stream.

The gas then enters a scrubber/steam generator vessel, where additional PW is introduced to further reduce the temperature to approximately 10 °C above the saturation point at operating pressure. This final stage raises the steam content of the product gas to approximately 94–95 vol %.

Sample probes extract product gas at each section of the process and feed it into moisture and gas analyzers to determine the composition of the product gas. Condensate from the moisture analyzers is collected to assess overall steam purity, while blowdown liquid samples from the quench and scrubber vessels as well as solid deposits formed within the system are collected to evaluate the fate of inorganic species present in the feedwater. All effluent streams are filtered, cooled, and depressurized prior to being sent for disposal.

The DCSG pilot plant facility was designed, constructed, installed, and commissioned at CanmetENERGY–Ottawa, as shown in Figure 4 along with its key operational parameters. The vessels were installed within a designated zone classified for explosive environments in a three-story building and integrated with utility connections for the SAGD water supply, oxygen/nitrogen/carbon dioxide supply, and natural gas.

**Figure 4:**
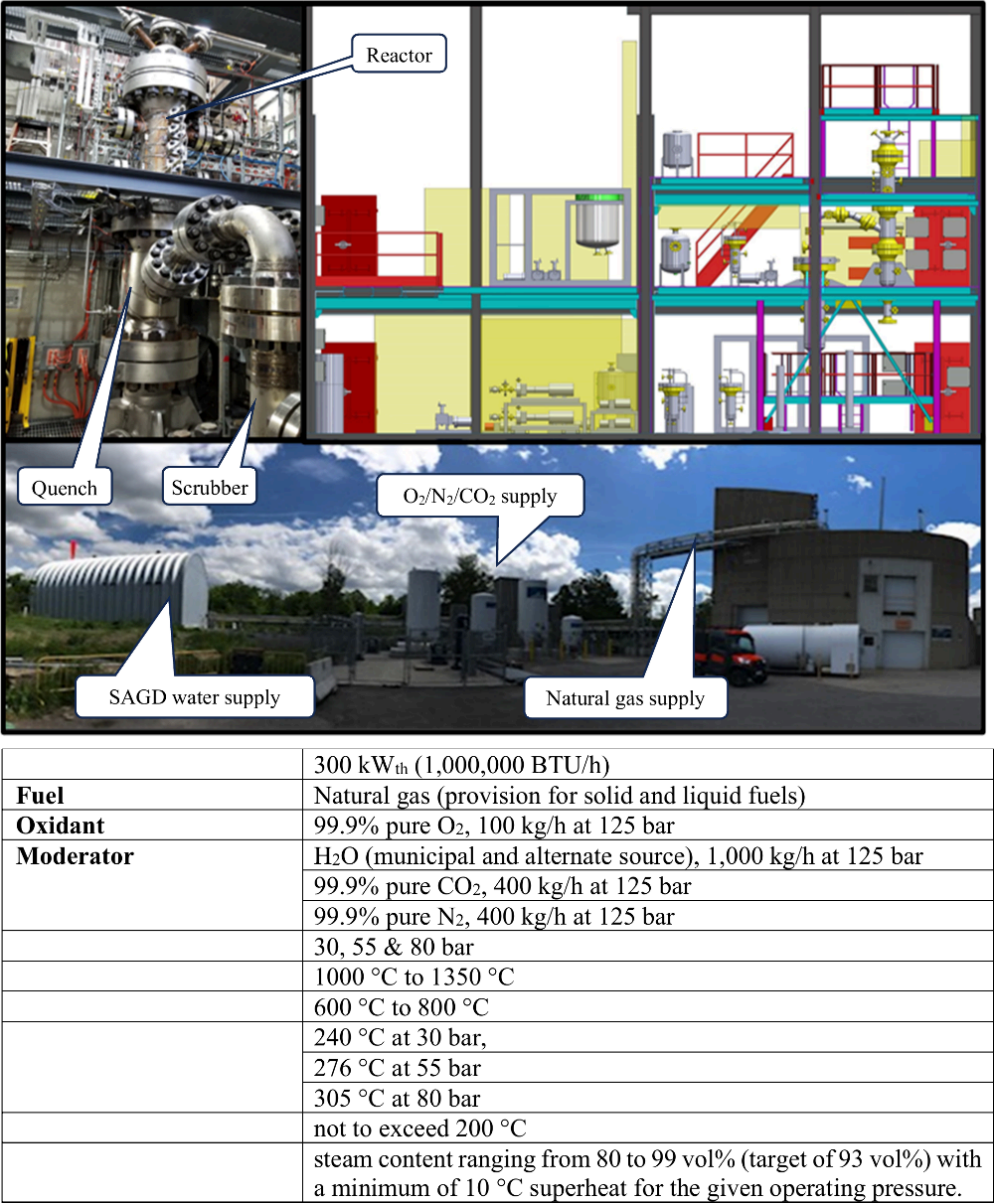
DCSG pilot plant at CanmetENERGY-Ottawa.

All control valves, pressure transmitters, flow transmitters, level sensors, and other instrumentation are automated via a centralized control system. This includes programmed sequences of operation and detailed logic describing how each system and subsystem responds to specific inputs such as temperature, pressure, level, flow, or alarms. The control station’s graphical interface was developed using ABB Freelance software.

The DCSG pilot plant is equipped with five operator stations, each with two monitors. The system is divided into 18 individual control screens. Typically, eight of the ten monitors are used for active control and operations, while the remaining two display graphical trends, such as refractory temperatures and supply system pressures.

### 2.1 Reactor, Quench, and Scrubber Specifications

Figure 5 illustrates the assembly of the reactor, quench, and scrubber sections, along with their internal configurations. The reactor vessel is constructed from a 12-in. prefabricated SS316L pipe (Schedule 160), lined with refractory material, insulation, and an active water-cooling jacket. The reactor has an internal diameter of 4 in. and a length-to-diameter ratio of 10, allowing for residence times of approximately 0.6 s at 30 barg and 1.5 s at 80 barg.

**Figure 5:**
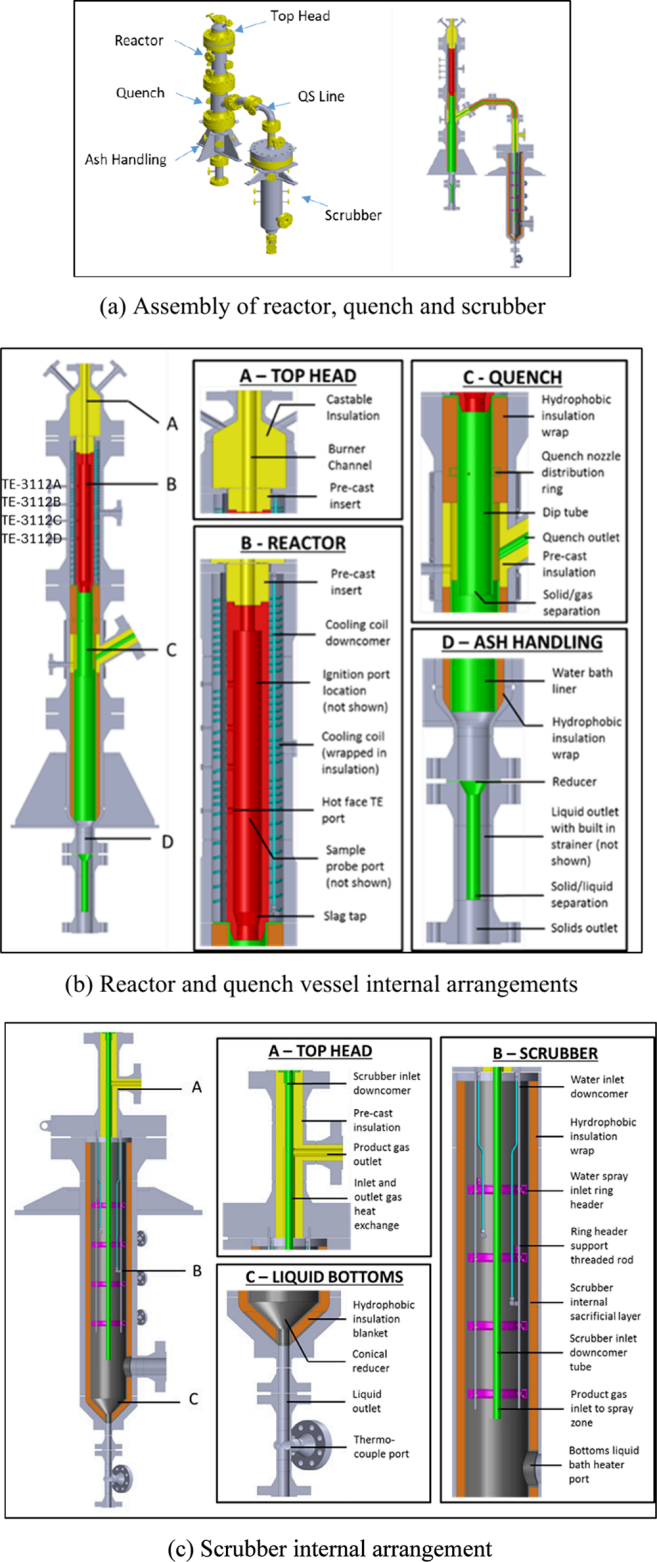
(a) Assembly of reactor, quench, and scrubber, (b) reactor and quench vessel internal arrangements, and (c) scrubber internal arrangement.

Positioned directly below the reactor, the quench vessel sprays water to reduce the product gas temperature from approximately 1350 to 600 °C. The water is atomized with an average droplet size of 100–150 μm^36^ to promote efficient cooling.

The scrubber employs a central downcomer that directs the incoming gas from the top of the vessel to the bottom, where it reverses direction and flows upward through a series of water sprays. Upon exiting the scrubber, the product gas undergoes slight superheating via an integrated heat exchanger located in the scrubber top-head. The scrubber is constructed from an 18 in. prefabricated SS316L pipe (Schedule 160), lined with insulation. Its internal diameter was designed to provide sufficient residence time to enable complete evaporation of the scrubbing water, with 8 s at 30 barg and 20 s at 80 barg. The droplet evaporation time was designed to be 1 s for a 150 μm droplet size, ensuring complete evaporation. Additionally, a heating coil is installed in the water bath at the bottom of the scrubber to preheat the vessel before startup, minimizing the time required to reach steady-state operation.

### 2.2 Burner Specifications

A single design concept was employed to develop three burners—each tailored for operation at a specific pressure level (30, 55, and 80 barg), as shown in Figure 6. Each burner features a central injector that premixes POW and natural gas to produce a fine mist with a relatively narrow spray angle of approximately 12 to 15 degrees. Surrounding this central atomizing injector are four pinhole oxygen injectors arranged circumferentially.

**Figure 6:**
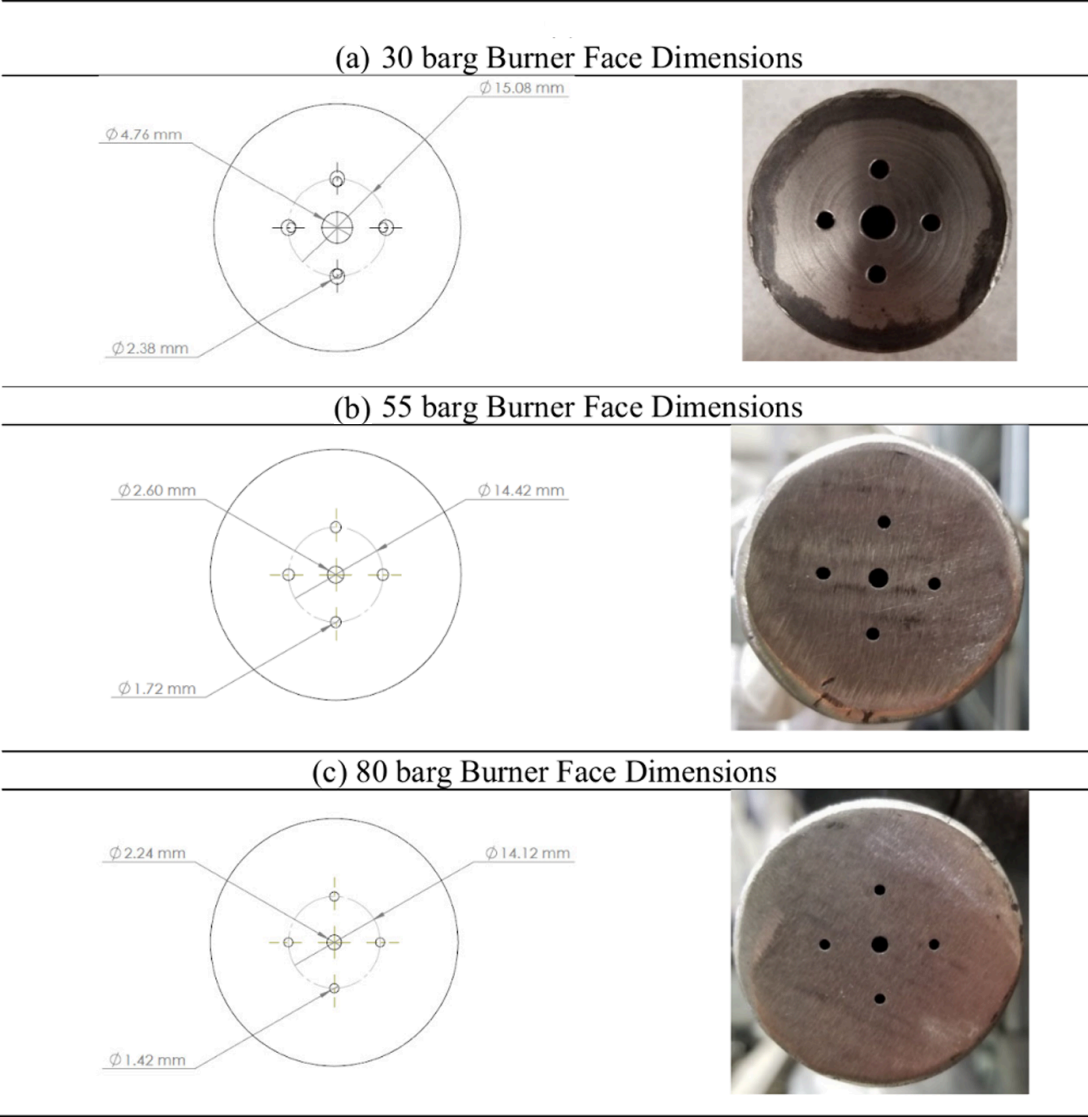
Burner for operating pressures of (a) 30, (b) 55, and (c) 80 barg.

This configuration enables external mixing of fuel and oxidant, ensuring that the flame anchors at a safe distance from the burner face to prevent overheating, an issue previously encountered with a pintle-style burner tested on liquid fuels during 15 barg tests.^35^ Additionally, the high-velocity oxygen jets serve to confine the spray path of the water droplets, minimizing wall impingement in the narrow reactor chamber. The injector orifice size was progressively reduced for higher operating pressures to maintain proper atomization and mixing characteristics.^36,37^

### 2.3 Experimental Cases

The performance of the pilot-scale DCSG system was evaluated using POW and PW sourced from an SAGD process. The compositions of POW and PW can vary, depending on the field operating conditions at the time of collection. The typical compositions of the water samples used in this study are summarized in Table 1. Values below detection limits are reported as zero, and elements present at insignificant concentrations are not included in the table. Prior to testing each case, composition analyses were performed again on the POW and PW samples.

**Table 1:** Typical Composition of POW/PW from SAGD Used for Testing the DCSG Pilot Plant

typical composition		POW	PW
Boron (total)	[mg/L]	18	17
Calcium (total)	[mg/L]	3	4
Copper (total)	[mg/L]	3	0
Iron (total)	[mg/L]	2	1
Potassium (total)	[mg/L]	23	21
Magnesium (total)	[mg/L]	0.5	1
Sodium (total)	[mg/L]	229	216
Sulfur (total)	[mg/L]	79	67
Silica (total)	[mg/L]	114	118
Total Suspended Solids	[mg/L]	75	135
Total Dissolved Solids	[mg/L]	1352	1274
Total Organic Carbon	[mg/L]	363	333

The DCSG system was rated for operation at up to 100 barg, and a series of experimental case studies were conducted to verify its performance under various target conditions. Tests were conducted at operating pressures of 80, 55, and 30 barg, using natural gas and oxygen as the combustion inputs. Key data collected during the experiments included the following: product gas composition (e.g., steam, CO_2_ and overall composition including impurities such as CO); burner ignition and stability; natural gas burnout; mineral deposition within the reactor and quench vessel; scrubber performance (including water acidity, solids removal, and impurity removal efficiency); minimum oxygen requirements to achieve complete combustion with minimal syngas formation; and system/material inspections conducted during and after operation. Steam samples were also collected and analyzed to characterize steam quality.

To support pilot-scale testing, process modeling and simulations were conducted under a range of test conditions. Data from the pilot-scale experiments were used to validate the simulation models, which will guide the development and design of a future demonstration-scale DCSG facility.

Table 2 provides an overview of the test cases. With POW injected into the reactor and PW used in the quench and scrubber, cases 1, 2, and 3 were conducted to evaluate operation in the temperature range from 1000 to 1250 °C in the reactor at 80 barg, including the behavior of inorganic components in the feedwater under each condition. Cases 4 to 6 were repeat tests at 80 barg for high and intermediate temperatures. A new refractory liner was used for each pair of repeat tests, then removed and cross-sectioned to assess whether slag properties or composition changed significantly and how they interacted with the refractory material to be presented in a subsequent study. Additional tests at 55 and 30 barg were conducted in cases 7 and 8 at intermediate temperatures.

**Table 2:** Experimental Cases for Testing the DCSG Pilot Plant with SAGD POW and PW

Case #	Objective	Target Reactor Pressure (barg)	Target Reactor Temp (°C)	Target Product Temp (°C)	Reactor Water injection	Quench Water injection	Scrubber Water injection
1	High Pres & Low Temp	80	1000	305	POW	PW	PW
2	High Pres & Int. Temp	80	1100	305	POW	PW	PW
3	High Pres & High Temp	80	1250	305	POW	PW	PW
4	High Pres & High Temp Repeat	80	1250	305	POW	PW	PW
5	High Pres & Int. Temp Repeat	80	1100	305	POW	PW	PW
6	High Pres & Int. Temp Repeat	80	1100	305	POW	PW	PW
7	Int. Pres & Int. Temp	55	1100	285	POW	PW	PW
8	Low Press & Int. Temp	30	1100	250	POW	PW	PW

### 2.4 Gas, Liquid, and Solids Sampling Locations in the Pilot-Scale DCSG Plant

In-process measurements were taken from all process streams, and samples were collected throughout the test runs as well as after shutdown. The locations for gas, liquid, and solid sampling are shown in Figure 7. Collected samples included: fuel and injected water, quench outlet gases (intermediate product), scrubber outlet gas (final product), as well as condensate, blowdown, and mineral residues from various locations (e.g., burner, liquid/gas bag filters, blowdown streams, and refractory liner). These samples were sent to both in-house and commercial characterization laboratories to evaluate the product gas composition, the fate of mineral matter, the composition of the blowdown streams, and material compatibility or interactions within the system.

**Figure 7:**
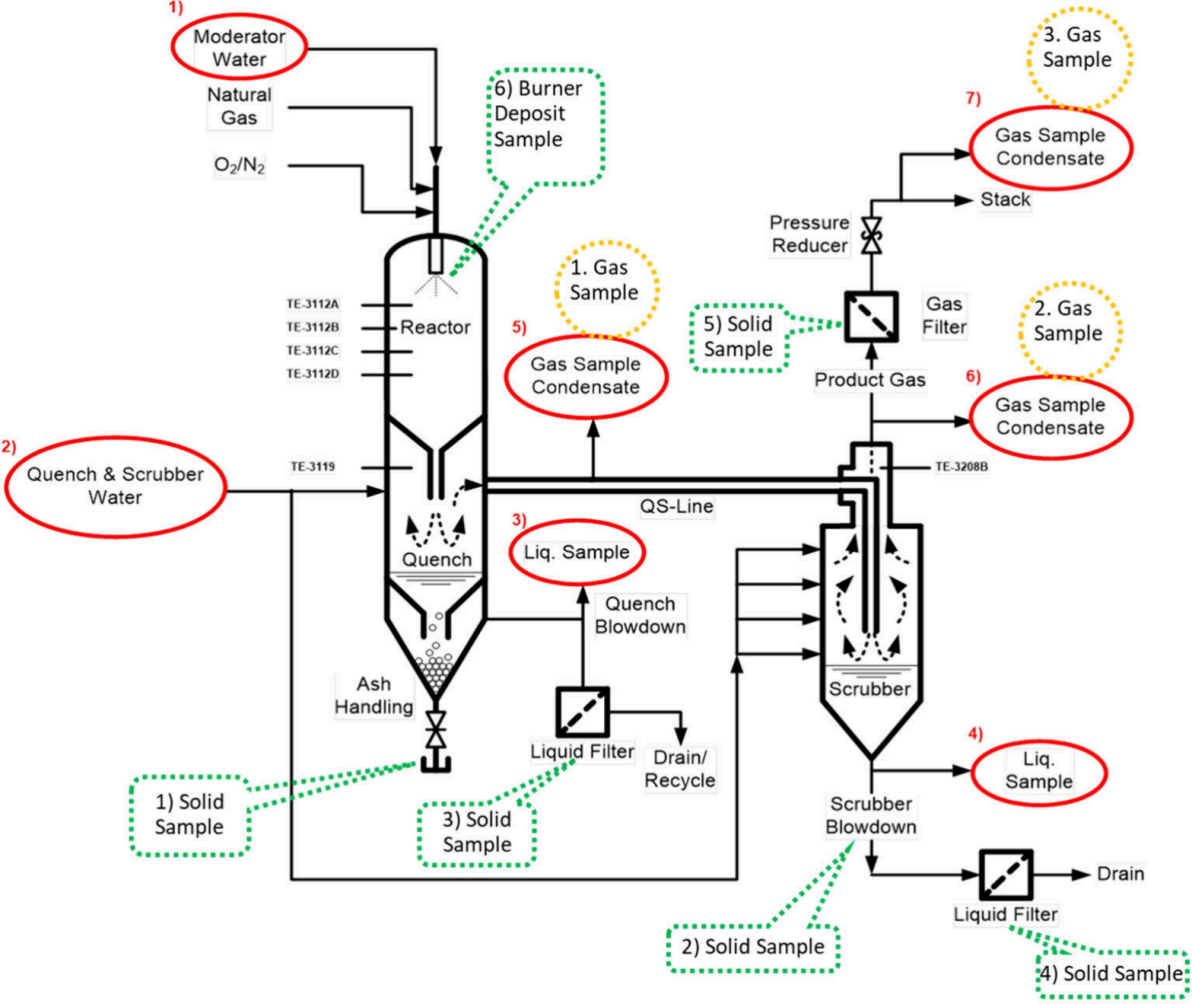
Gas sampling locations (3, orange), liquid sampling locations (7, red), and solid sampling locations (6, green) in the DCSG pilot plant.

## 3 Case Study Results and Discussion

### 3.1 Operation and Performance Parameters

The performance parameters for the eight test cases using SAGD water are summarized in Table 3. In cases 1 through 6, where the target operating conditions were 80 barg and 1000–1250 °C, the pilot-scale DCSG system successfully operated at pressures ranging from 76 to 79 barg and temperatures between 1016 and 1236 °C. The resulting product gas had steam contents between 76 and 95 vol %, and the actual product gas output achieved 73 to 95% of the theoretically calculated maximum yield. In case 7, which targeted 55 barg and 1100 °C, the system operated at an average pressure of approximately 60 barg and a temperature of 1098 °C, producing product gas with a steam content of ∼87 vol % and a gas yield of 89%. In case 8, targeting 30 barg and 1100 °C, the system achieved an average operating pressure and temperature of 29 barg and 1057 °C, respectively, with the product gas containing ∼89 vol % steam and a yield of 83%.

**Table 3:** Key Experimental Performance Parameters for Cases 1 to 8

performance parameters	Case 1	Case 2	Case 3	Case 4	Case 5	Case 6	Case 7	Case 8
Average Steady-State Operating Pressure, barg	78.2 ± 1.9	78.9 ± 2.1	76.4 ± 0.8	78.2 ± 0.8	77.1 ± 0.7	76.7 ± 1.1	59.6 ± 1.4	29.3 ± 0.8
Average Steady-State Operating Temperature, °C	1016 ± 13	1135 ± 20	1216 ± 15	1236 ± 17	1103 ± 5	1129 ± 12	1098 ± 22	1057 ± 70
Average Steady-State Firing Rate, KW	297 ± 32	277 ± 51	291 ± 17	284 ± 18	296 ± 5	296 ± 13	297 ± 7	259 ± 26
POW Injection Rate, kg/h	160 ± 23	133 ± 27	141 ± 9	137 ± 8	160 ± 6	156 ± 7	161 ± 3	153 ± 6
PW Injection Rate (Quench), kg/h	36 ± 8	4 ± 5	40 ± 10	44 ± 6	32 ± 5	21 ± 5	57 ± 7	14 ± 1
PW Injection Rate (Scrubber), kg/h	60 ± 15	127 ± 36	143 ± 22	108 ± 6	97 ± 7	119 ± 8	85 ± 11	50 ± 8
Product Gas Rate, kg/h	342 ± 29	269 ± 47	320 ± 26	338 ± 12	362 ± 10	349 ± 12	390 ± 14	294 ± 10
Product Gas Rate Yield (vs Theoretical), %	77.62	73.68	94.38	87.62	83.12	93.18	89.92	82.96
Product Gas Steam Content, vol %	94 ± 2	89 ± 1	91 ± 1	92 ± 1	92 ± 1	91 ± 1	87 ± 4	89 ± 1
Average Steady-State Product Gas Temperature, °C (Saturation T, °C)	302 ± 6 (293)	324 ± 5 (294)	315 ± 4 (291)	310 ± 2 (293)	313 ± 2 (293)	313 ± 3 (292)	293 ± 3 (275)	252 ± 3 (234)
Quench Blowdown, kg/h	0.09	0.00	0.02	0.01	0.78	0.07	0.01	0.47
Scrubber Blowdown, kg/h	13.38	89.36	101.87	48.71	26.91	46.90	13.79	3.57
Duration, hours	7	3	6	5.5	6	6.5	7	6

The operation of the DCSG unit demonstrated excellent overall resilience across a wide range of operating conditions and exhibited the ability to quickly recover from major process disturbances. The system produced a highly uniform temperature profile, as shown in Figure 8 and Figure 9. The temperature gradient along the length of the reactor’s refractory liner (hot-face) was small, as indicated by the four temperature sensors TE-3112A, B, C, and D, which are vertically spaced along the reactor, as shown in Figure 5 (b). The in-house designed and manufactured ignition system achieved repeatable and reliable ignition of the pilot unit at the desired operating pressure without the need to slowly ramp up the vessel pressure after ignition. Reignition after a flame disturbance or automated shutdown was also possible using the refractory liner as a hot-surface ignition source. This radiant heat interlock was a built-in feature of the burner management system, which would allow the operators to reignite the system without the use of the ignitor probe if any of the temperatures on the refractory liner thermocouples are reading above 760 °C.

**Figure 8:**
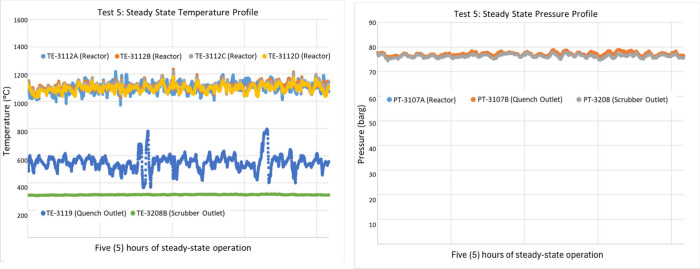
Steady-state temperature/pressure profile with a target reactor temperature of 1100 °C.

**Figure 9:**
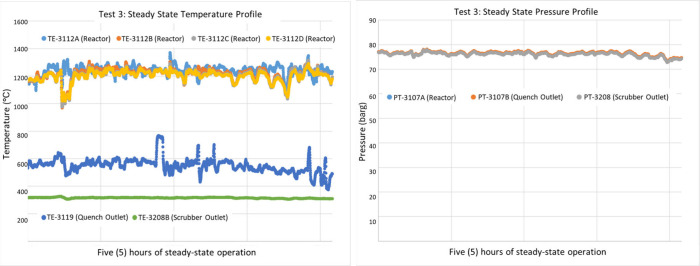
Steady-state temperature/pressure profile with a target reactor temperature of 1250 °C.

### 3.2 Solids Depositions

After each test, the vessels were cooled and opened for solids collection. At the target operating pressure of 80 barg, each test revealed deposition of solids on the burner face, as shown in Figure 10. These deposits were easily flaked off with some displaying blue and green hues. During the tests for Cases 2 and 6, system shutdowns occurred, likely due to these deposits partially plugging the oxygen nozzles and causing a drop in O_2_ flow. When a pressure drop across the burner face was detected, the reactor temperature was deliberately raised above 1300 °C by increasing the natural gas and oxygen flow rates in an attempt to thermally remove the obstruction. This operational approach was occasionally successful but inconsistent. When unsuccessful, a shutdown would occur, and an attempt to reignite the reactor would ensue. If reignition was unsuccessful, the test would be terminated and repeated or if the test had run for a sufficient period of time that allowed for sufficient samples to be collected then deemed completed. During these excursions, temperatures and emissions would spike or drop temporarily until a steady state was re-established.

**Figure 10:**
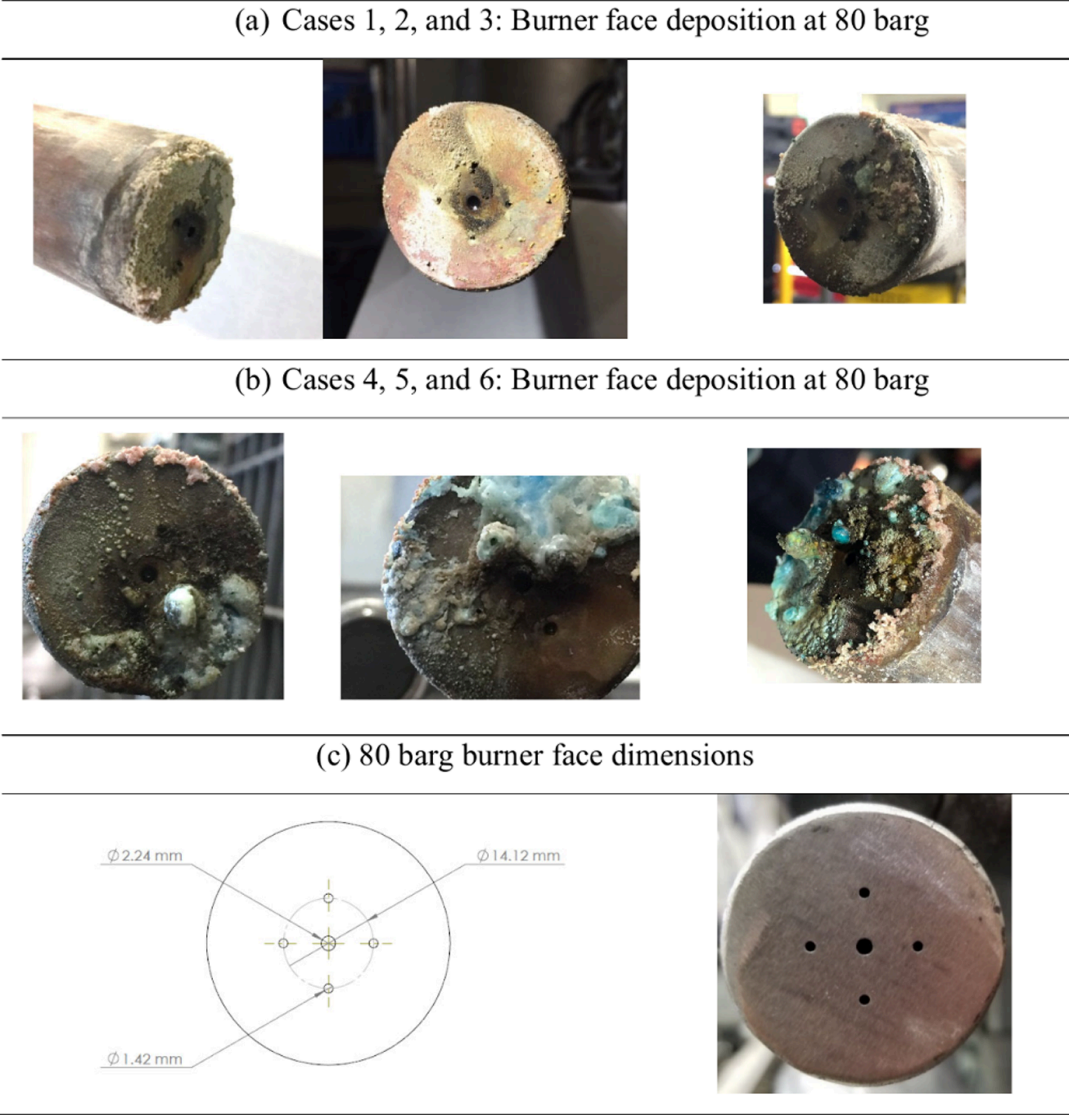
(a, b) Burner face deposition at 80 barg.

At the intermediate target pressure of 55 barg and the low pressure of 30 barg, burner face deposition was significantly less pronounced compared to the 80 barg operation, i.e., moderate at 55 barg and minimal at 30 barg, as shown in Figure 11. This trend is most likely attributed to changes in the gas density and their impact on recirculation behavior. Although burner velocity and mass flow were held constant across all pressure conditions, maintaining constant momentum, the denser medium at 80 barg promotes stronger recirculation zones. These zones can trap solid particles and carry them back toward the burner face, increasing the deposition. In contrast, at lower pressures (30 and 55 barg), the reduced gas density weakens recirculation, allowing water droplets to travel further before breaking up into smaller particles. These particles are less likely to be re-entrained and deposited, thereby reducing fouling at the burner face.

**Figure 11:**
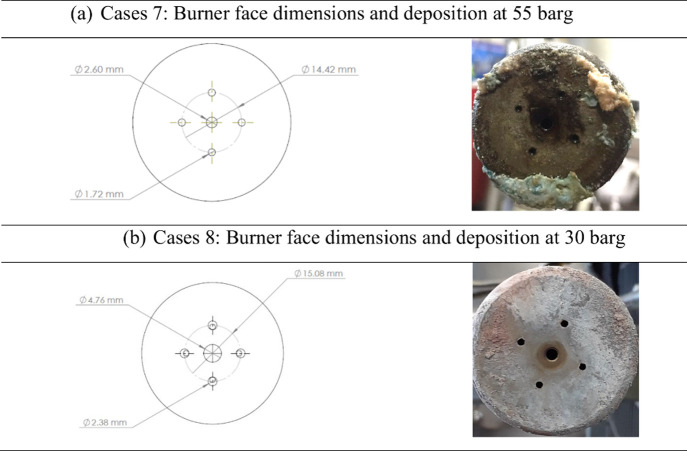
(a, b) Burner face depositions at 55 and 30 barg.

To conclusively determine the relationship between the reactor pressure and burner deposition, additional tests across a full range of pressures using various water types would be required. It is hypothesized that the observed reduction in deposition is primarily due to changes in the bulk velocity of injectants rather than reactor pressure alone. Lower reactor pressure results in a lower gas density, thereby increasing gas velocity. Additionally, the 55-barg burner produced larger droplets than the 80-barg burner, leading to increased droplet momentum due to the higher mass and velocity. This enhanced momentum likely carried inorganic matter further downstream, reducing the likelihood of recirculation and accumulation on the burner face.

Each test also resulted in the deposition of solids on other locations in the DCSG unit. Large quantities of glassy droplets of various colors were found in the quench bottoms, as shown in Figure 12.

**Figure 12:**
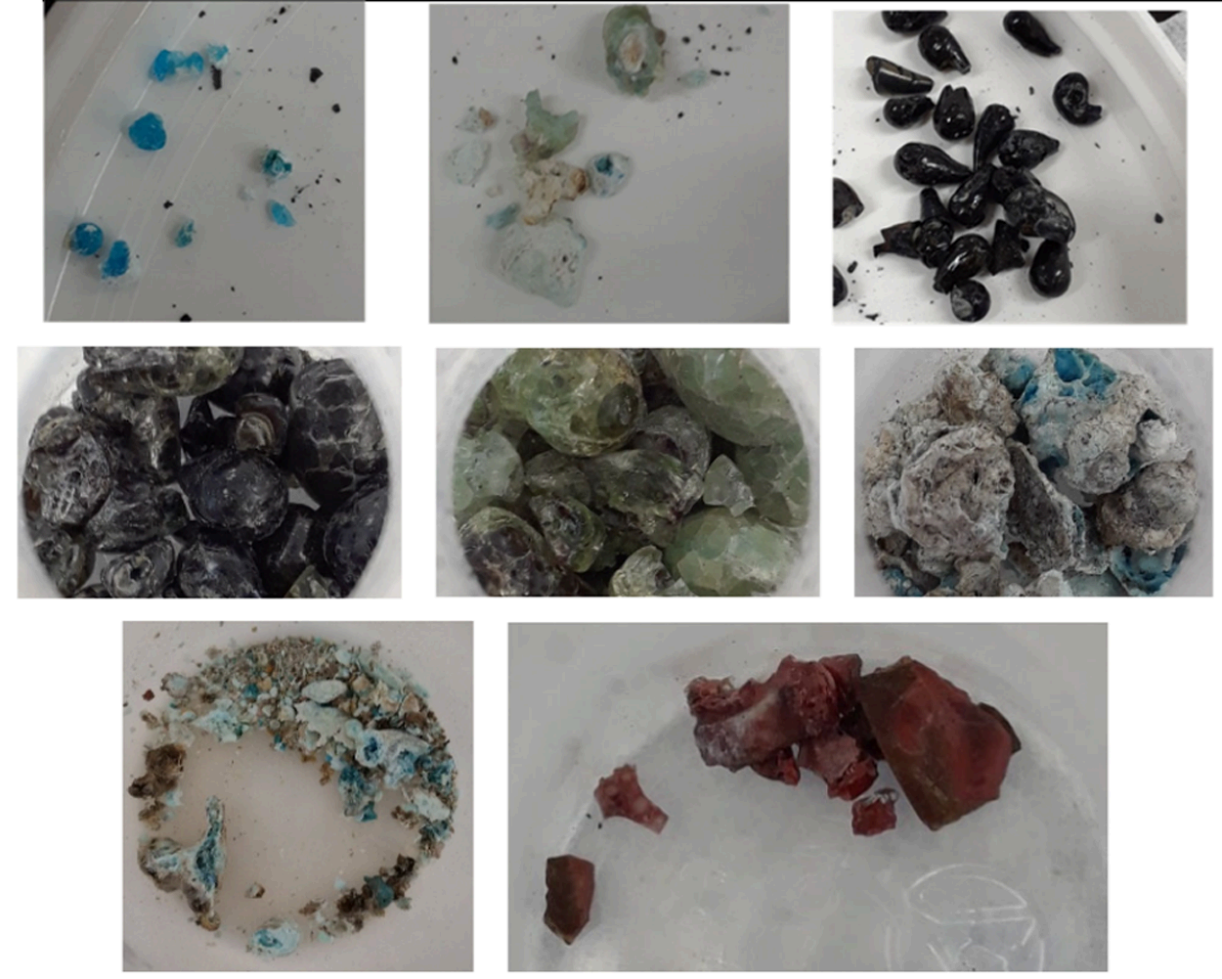
Glassy solid droplets collected from the quench blowdown.

Solids collected from the scrubber vessel blowdown, as shown in Figure 13, were small, flakey, and very brittle.

**Figure 13:**
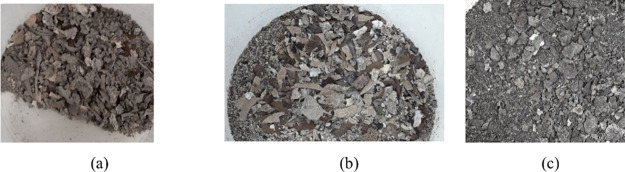
Scrubber Blowdown Deposition from 80 barg operation at targeted temperature: a) 1000 °C, b) 1100 °C, c) 1250 °C.

### 3.3 Gas, Liquid and Solids Analysis

For the composition analysis of various samples, all liquid analyses (water, condensate, etc.) were performed by SGS Canada Inc., a commercial laboratory, while solid sample analyses and gas analyses (natural gas) were conducted at the CanmetENERGY characterization laboratory. Continuous in-system gas measurements were carried out by using Horiba Model 510 gas analyzers.

The basic combustion reaction of natural gas with high-purity oxygen remained consistent across all of the test cases. Product gas samples were collected at locations 1, 2, and 3, as shown in Figure 7. In all cases, the product gas was rich in steam and CO_2_. Summary data at 80 barg, averaged from tests conducted at 1100 °C (Cases 2, 5, and 6) and 1250 °C (Cases 3 and 4), are provided in Table 4. Trace amounts of H_2_, CH_4_, and N_2_ were below the detection limits.

**Table 4:** Product Gas Analysis for Cases at 80 barg^a^

	quench, Loc. 1		scrubber, Loc. 2		back-end, Loc. 3	
product gas analysis	1100 °C average of cases 2, 5 and 6	1250 °C average of cases 3 and 4	1100 °C average of cases 2, 5 and 6	1250 °C average of cases 3 and 4	1100 °C average of cases 2, 5 and 6	1250 °C average of cases 3 and 4
O_2_ (vol % dry)	1.02	1.28	1.37	1.19	1.92	2.99
CO_2_ (vol % dry)	89.84	88.60	89.71	89.39	87.01	87.30
CO (ppm dry)	1938	1919	1905	2114	1899	1750
H_2_O (vol %)	89.79	86.32	91.94	92.98	90.70	92.39

a Note: Continuous monitoring of O_2_, CO_2_, and CO was performed using Horiba Model 510 gas analyzers.

Liquid samples were collected at locations 1–7 in Figure 7. At the beginning of the test, the blowdown vessels were initially filled with city water. Throughout the test, blowdown was generated and continuously introduced into the vessel without active mixing. As a result, the blowdown samples were likely nonhomogeneous. The values presented in Table 5 are averaged from the tests conducted at 80 barg (1100 and 1250 °C; Cases 2, 3, 4, 5, and 6) over the test duration. The blowdown water values represent the average change in concentration relative to the injected water supplies, with Loc. 3 compared to Loc. 1, and Loc. 4 compared to Loc. 2, respectively.

**Table 5:** Liquid Analysis at 80 barg^a^

		injected water										
		POW		PW		change in blowdown concentration relative to injected water				product gas cond.		
		Loc. 1 reactor		Loc. 2 quench		Loc. 3 quench		Loc. 4 scrubber		Loc. 7 stack		
liquid analysis		1100 °C	1250 °C	1100 °C	1250 °C	1100 °C	1250 °C	1100 °C	1250 °C	1100 °C	1250 °C	city water
Boron (total)	[mg/L]	20	15	19	14	11	3	28	–9	7	4	0
Calcium (total)	[mg/L]	3	3	4	3	2	3	–2	0	0	0	4
Copper (total)	[mg/L]	3	2	0	0	0	0	0	1	0	0	0
Iron (total)	[mg/L]	2	1	2	1	1	1	0	1	0	0	0
Potassium (total)	[mg/L]	25	20	23	18	18	5	40	–15	0	0	0
Magnesium (total)	[mg/L]	1	0	1	1	0	0	0	0	0	0	1
Sodium (total)	[mg/L]	255	191	252	163	168	41	304	–92	5	2	23
Sulfur (total)	[mg/L]	87	67	75	55	33	13	93	–42	7	4	9
Silica (total)	[mg/L]	106	125	106	137	98	86	58	–56	20	64	30
Reactive Silica	[mg/L]	193	155	173	138	147	26	375	–60	1	1	5
Organic Carbon (Total)	[mg/L]	345	391	314	363	112	–7	146	100	67	61	4
Suspended Solids (Total)	[mg/L]	73	77	123	153	11	12	46	58	0	0	0
Dissolved Solids (Total)	[mg/L]	1400	1280	1317	1210	1121	212	1275	–1175	0	22	96
Floride	[mg/L]	3	3	3	3	2	0	5	1	1	1	1
Sulfate	[mg/L]	52	53	41	57	61	37	559	90	1	0	24
Chloride	[mg/L]	113	130	98	110	55	10	160	0	1	2	6
Nitrite (as N)	[mg/L]	0	0	0	0	2	0	1	0	0	0	0
Nitrate (as N)	[mg/L]	0	0	0	0	2	0	0	0	0	0	0
Phosphorus (total reactive)	[mg/L]	1	0	1	0	0	0	0	0	0	0	0
Sulfide	[mg/L]	1	1	1	1	0	–2	–1	–1	0	0	0

a Values represent the average of cases 2, 5, and 6 for 1100 °C, and cases 3 and 4 for 1250 °C. Blowdown values are relative to the injected water: Loc. 3 vs Loc. 1, and Loc. 4 vs. Loc. 2. Note: Liquid analyses were conducted by SGS Canada Inc.

For trace metal analysis, all samples underwent digestion using hydrofluoric acid. Consequently, silica concentrations appear artificially low due to volatilization of silicon tetrafluoride during the digestion process. Values below detection limits are reported as zero, and elements present at insignificant concentrations are not included in the table. The product gas was condensed, and the condensate was analyzed. The condensate quality was reasonably clean compared to city water quality included in Table 5.

Solid samples were collected from locations 1 to 6 in Figure 7, with results from locations 1 and 2 summarized in Table 6. These samples showed a high fraction of sodium relative to other trace metals. Excess solid samples from the quench bottom were combined at the end of the test series for toxicity characteristic leaching procedure (TCLP) analysis; results are presented in Table 7. Most metals and organics are below TCLP limits, except chromium (7.55 mg/L) which is above the U.S. EPA TCLP hazardous waste threshold (5 mg/L).

**Table 6:** Solids Analysis from Quench and Scrubber Blowdown Streams^a^

		case				case	
		POW/PW, (80 barg, 1250 °C)				POW/PW, (80 barg, 1250 °C)	
element		quench, Loc. 1	scrubber, Loc. 2	element		quench, Loc. 1	scrubber, Loc. 2
SiO_2_	[wt %]	13.58	61.08	BaO	[wt %]	0.01	0.04
Al_2_O_3_	[wt %]	1.39	6.15	SrO	[wt %]	0.00	0.02
Fe_2_O_3_	[wt %]	59.41	7.5	V_2_O_5_	[wt %]	0.07	0.02
TiO_2_	[wt %]	0.01	0.15	NiO	[wt %]	5.49	0.32
P_2_O_5_	[wt %]	0.04	0.20	MnO	[wt %]	1.27	0.13
CaO	[wt %]	1.98	1.55	Cr_2_O_3_	[wt %]	10.24	0.63
MgO	[wt %]	0.07	0.34	CuO	[wt %]	0.24	0.42
SO_3_	[wt %]	0.14	1.45	ZnO	[wt %]	0.02	0.04
Na_2_O	[wt %]	4.68	5.08	LOI	[wt %]	0.88	14.10
K_2_O	[wt %]	0.4	0.79	**Sum**	**[wt %]**	**99.93**	**100.00**

a Notes: solid analyses were conducted by the CanmetENERGY characterization laboratory.

**Table 7:** Toxicity Characteristic Leaching Procedure (TCLP) Analysis Results from Combined Quench Bottom Solids Collected in the DCSG Pilot Plant^a^

composition		quench bottom solids	composition		quench bottom solids
Final pH	no unit	5.45	Vinyl chloride	[mg/L]	<0.008
Mercury	[mg/L]	<0.00001	Dichloromethane	[mg/L]	<0.02
Arsenic	[mg/L]	<0.01	Chloroform	[mg/L]	<0.02
Silver	[mg/L]	<0.08	Benzene	[mg/L]	<0.02
Barium	[mg/L]	0.445	Trichloroethylene	[mg/L]	<0.02
Boron	[mg/L]	1.57	Tetrachloroethene	[mg/L]	<0.02
Cadmium	[mg/L]	<0.001	Monochlorobenzene	[mg/L]	<0.02
Chromium	[mg/L]	7.55	Carbon tetrachloride	[mg/L]	<0.008
Lead	[mg/L]	0.019	1,2-Dichlorobenzene	[mg/L]	<0.02
Selenium	[mg/L]	0.05	1,4-Dichlorobenzene	[mg/L]	<0.02
Uranium	[mg/L]	0.1	1,2-Dichloroethane	[mg/L]	<0.02
Methylethylketone	[mg/L]	<0.8	1,1-Dichloroethylene	[mg/L]	<0.02

a Notes: solid analyses were conducted by the CanmetENERGY characterization laboratory.

## 4 Conclusion

The DCSG system demonstrated stable operation across a wide range of operating conditions with SAGD water to achieve a pressurized steam-rich flue gas composed of approximately 90% steam, balanced by CO_2_ and trace gases. It maintained a uniform temperature profile along the reactor length. The ignition system reliably achieved ignition at the target pressures, including successful hot reignition after flame disturbances or automatic shutdowns.

During the SAGD POW/PW tests, two primary issues were observed: (1) deposition of inorganic material on the burner face under 80 barg conditions and (2) disturbances in POW feed supply at the same pressure. These issues led to fluctuations in flow rate—both surges and sudden drops. Water surges rapidly reduced the reactor temperature and increased the reactor pressure due to excess steam generation, while sudden flow drops caused temperature spikes and pressure drops due to decreased steam production. These issues were largely mitigated at lower operating pressures (55 and 30 barg). Cold-flow testing revealed that the mean spray droplet diameter was ∼25 μm at 80 barg compared to ∼45 μm at 55 and 30 barg. The smaller droplets at high pressure likely evaporated closer to the burner face, allowing inorganic material to become entrained in recirculating gases and deposit on the burner rather than being carried further downstream. Lower injection gas velocities at higher pressures may have exacerbated this effect. While these issues might be less severe at a commercial scale, they underscore the importance of an optimized burner design for high-pressure DCSG applications.

Condensate samples from the product steam exhibited very low solid content, suggesting minimal scaling risk for steam transport and injection lines in SAGD facilities. The product gas quality was sufficient for heat integration, including use in condensing heat exchangers. Trace amounts of O_2_, CO, H_2_, and CH_4_ were detected and should be considered in safety assessments for operation.

The combustion and scrubbing processes effectively separated solids from SAGD POW/PW. Blowdown solids handling for commercial-scale operation could adopt proven industrial methods, such as pressurized fluidized bed combustion ash handling systems, which manage both bottom ash and fly ash, often using a combination of mechanical and pneumatic methods.^38^

It should be noted that these pilot tests had a maximum duration of 7 h. Long-term operation introduces greater uncertainty. As such, a field demonstration facility represents the logical next step prior to the commercial deployment of DCSG technology.

As compared with conventional boiler steam generation, DCSG has the advantages of being smaller and more transportable, having high energy efficiency, the ability to use somewhat lower quality water, and requiring significantly less capital expense for water treatment.

## Abbreviations

DCSG: direct contact steam generation
HiPrOx: high pressure oxy-fired
NCG: noncondensable gas
OTSG: once-through steam generator
POW: produced oily water
PW: produced water
SAGD: steam-assisted gravity drainage
TDS: total dissolved solids
TOC: total organic carbon
BFW: boiler feedwater
BBD: boiler blowdown
WLS/HLS: warm or hot lime softening

## References

[1] IEA , Net Zero Roadmap: A Global Pathway to Keep the 1.5 °C Goal in Reach – 2023 Update, September 2023, accessed August 1, 2025, https://www.iea.org/reports/net-zero-roadmap-a-global-pathway-to-keep-the-15-0c-goal-in-reach.

[2] DOE. DOE Announces $33 Million to Deploy Solar Technologies to Decarbonize America’s Industrial Sector, https://www.energy.gov/articles/doe-announces-33-million-deploy-solar-technologies-decarbonize-americas-industrial-sector?utm_source=chatgpt.com, accessed May 30th, 2025.

[3] Okamoto K., Asanuma H., Ishibashi T., Yamaya Y., Saishu H., Yanagisawa N., Mogi T., Tsuchiya N., Okamoto A., Naganawa S., Ogawa Y., Ishitsuka K., Fujimitsu Y., Kitamura K., Kajiwara T., Horimoto S., Shimada K. (2019). Geological and Engineering Features of Developing Ultra-High-Temperature
Geothermal Systems in the World. Geothermics.

[4] Song X., Li G., Huang Z., Shi Y., Wang G., Song G., Xu F. (2023). Review of high temperature
geothermal drilling and exploitation technologies. Gondwana Research.

[5] Akhigbe E. E. (2025). Advancing
geothermal energy: A review of technological developments and environmental
impacts. Gulf Journal of Advance Business Research.

[6] Mignogna D., Szabo M., Ceci P., Avino P. (2024). Biomass Energy and
Biofuels: Perspective, Potentials, and Challenges in the Energy Transition. Sustainability.

[7] Ashabi A., Mostafa M., Hryshchenko A., Bruton K., O’sullivan D. T.J. (2025). Assessing
Power-to-Heat Technologies for Industrial Electrification: A Multi-Criteria
Decision Analysis Approach. Energy Conversion
and Management: X.

[8] Ciambellotti A., Frate G. F., Baccioli A., Desideri U. (2024). High-Temperature
Heat Pumps for Electrification and Cost-Effective Decarbonization
in the Tissue Paper Industry. Energies.

[9] Shokri A., Sanavi Fard M. (2023). Principles,
Operational Challenges, and Perspectives
in Boiler Feedwater Treatment Process. Environmental
Advances.

[10] Guo D., Wang H., Fu P., Huang Y., Liu Y., Lv W., Wang F. (2018). Diatomite
Precoat Filtration for Wastewater Treatment:
Filtration Performance and Pollution Mechanisms. Chem. Eng. Res. Des..

[11] Piccioli M., Aanesen S. V., Zhao H., Dudek M., Øye G. (2020). Gas Flotation
of Petroleum Produced Water: A Review on Status, Fundamental Aspects,
and Perspectives. Energy Fuels.

[12] Shahid M. K., Pyo M., Choi Y. (2017). Carbonate
Scale Reduction in Reverse Osmosis Membrane
by CO_2_ in Wastewater Reclamation. Membr. Water Treat..

[13] Mohammadtabar F., Pillai R. G., Khorshidi B., Hayatbakhsh A., Sadrzadeh M. (2019). Efficient Treatment of Oil Sands
Produced Water: Process
Integration Using Ion Exchange Regeneration Wastewater as a Chemical
Coagulant. Sep. Purif. Technol..

[14] Vand, Z. S. ; Sallamie, N. SAGD produced water treatment: technologies and challenges. APEGA Conference, April 2019, Calgary.

[15] Alamatsaz A., Moore R., Mehta S., Ursenbach M. (2011). Experimental
investigation of in-situ combustion at low air fluxes. J. Can. Pet Tech.

[16] Clements, B. High pressure direct contact oxy-fired steam generator, Patent CA 2744825 C, May 05, 2015.

[17] Yadav S., Mondal S.S. (2022). A Review on the Progress and Prospects of Oxy-Fuel
Carbon Capture and Sequestration (CCS) Technology. Fuel.

[18] Clements, B. ; Pomalis, R. ; Zheng, L. ; Herage, T. Optimization of High Pressure Oxy-Fuel Combustion Process for Power Generation and CO_2_ Capture. In 35th International Technical Conference on Clean Coal and Fuel Systems; Clearwater, Florida, USA, 2010.

[19] Clements, B. ; Pomalis, R. ; Zheng, L. ; Herage, T. High pressure oxy-fuel (HiPrOx) combustion systems. In Oxy-Fuel Combustion for Power Generation and Carbon Dioxide (CO_2_) Capture; Zheng, L. , Ed.; Woodhead Publishing Limited, 2011.

[20] Talei S., Fozer D., Varbanov P. S., Szanyi A., Mizsey P. (2024). Oxyfuel Combustion Makes Carbon Capture More Efficient. ACS Omega.

[21] Carrasco-Maldonado F., Sporl R., Fleiger K., Hoenig V., Maier J., Scheffknecht G. (2016). Oxy-fuel combustion technology for
cement production
– State of the art research and technology development. International Journal of Greenhouse Gas Control.

[22] Makwana A. (2022). Decarbonization of Secondary Aluminum Melting: Oxy-Fuel
Combustion
and Low-Carbon Intensity Fuels. Light Metal
Age.

[23] Butler R. M. (1994). Steam-assisted
gravity drainage: Concept, development, performance and future. Journal of Canadian Petroleum Technology.

[24] Cairns, P. E. High Pressure Oxy-fired (HiPrOx) Direct Contact Steam Generation (DCSG) for Steam Assisted Gravity Drainage (SAGD) Application. Thesis submitted to the Faculty of Graduate and Postdoctoral Studies In partial fulfillment of the requirements for the M.A.Sc. degree in Chemical Engineering, University of Ottawa, 2013.

[25] Hajinasiri, J. Treatment of Steam Assisted Gravity Drainage Produced Water Using Polymeric Membranes. Thesis for Master of Science, 2015, 10.7939/R37941299.

[26] Li J., How Z. T., Zeng H., Gamal El-Din M. (2022). Treatment
Technologies for Organics and Silica Removal in Steam-Assisted Gravity
Drainage Produced Water: A Comprehensive Review. Energy Fuels.

[27] Choi Y., Kim Y., Woo Y. C., Hwang I. (2023). Water Management and Produced Water
Treatment in Oil Sand Plant: A Review. Desalination.

[28] Guha
Thakurta S., Maiti A., Pernitsky D. J., Bhattacharjee S. (2013). Dissolved Organic Matter in Steam Assisted Gravity
Drainage Boiler Blow-Down Water. Energy Fuels.

[29] Courtesy: Dr. Subir Bhattacharjee, Water Use in Oil Sands Industry, Overview – Advanced Water Research Lab, accessed October 3, 2025.

[30] Butler R. M., Jiang Q., Yee C. T. (2001). Steam and
Gas Push (SAGP)-4;Recent
Theoretical Developments and Laboratory Results Using Layered Models. Journal of Canadian Petroleum Technology.

[31] Nasr, T. N. , Brown, D. A. , Steam-Gas-Solvent (SGS) process for recovery of heavy crude oil and bitumen. US Patent 8474531. July 2013.

[32] Kim S., Shin H., Park C., Chen Z., Lee K. (2025). A Review of
Design Factors in Steam and Gas Push for Eco-Friendly Oil Sands Production
and Its Field Application in Canada. Journal
of Petroleum Exploration and Production Technology.

[33] Wang C., Liu P., Wang F., Atadurdyyev B., Ovluyagulyyev M. (2018). Experimental
Study on Effects of CO_2_ and Improving Oil Recovery for
CO_2_ Assisted SAGD in Super-Heavy-Oil Reservoirs. J. Pet. Sci. Eng..

[34] Beaton M. L., Mashhadi N., Dominato K. R., Maguire T. J., Rupert K. D., Mundle S. O.C. (2022). Monitoring CO_2_ Injection and Retention in
Steam-Assisted Gravity Drainage (SAGD) Operations. J. Pet. Sci. Eng..

[35] Cairns P., Clements B., Hughes R., Herage T., Zheng L., Macchi A., Anthony E. (2015). High-Pressure Oxy-Firing
(HiPrOx)
of Fuels with Water for the Purpose of Direct Contact Steam Generation. Energy Fuels.

[36] Geddis P., Corber A. (2020). Effervescent Spray
Measurement In An 80-Barg Cold-Flow
Test Facility. Atomization and Sprays.

[37] Geddis, P. . DCSG phase 2, sub-program 5 - Burner design and computational fluid dynamics validation, CanmetENERGY-Ottawa report, 2020.

[38] Babcock & Wilcox , B&W’s Allen-Sherman-Hoff bottom ash handling systems, https://www.babcock.com/home/environmental/a-s-h-material-and-ash-handling/ash-handling-systems#, accessed June 30, 2025.

